# Association between vitamin D and risk of cardiovascular disease in Chinese rural population

**DOI:** 10.1371/journal.pone.0217311

**Published:** 2019-05-23

**Authors:** Teng Wang, Hualei Sun, Huina Ge, Xinxin Liu, Fei Yu, Han Han, Jun Wang, Wenjie Li

**Affiliations:** Department of Nutrition and food hygiene, college of Public health of Zhengzhou University, Zhengzhou, China; Beijing Key Laboratory of Diabetes Prevention and Research, CHINA

## Abstract

Previous studies have suggested that vitamin D is associated with cardiovascular disease (CVD), however, the relationship between vitamin D levels and CVD risk is still unclear. The purpose of this study was to assess the relationship of serum concentration of 25-hydroxyvitamin D (25(OH)D) with CVD in rural residents of Henan province of China. Basic information and medical history were gathered through face-to-face surveys from July 2013 to August 2015, and biochemical indicators were gathered in a laboratory setting. Logistic and restricted cubic splines regression analyses were used to estimate odd ratios (ORs) and 95% confidence intervals (95%CI) of CVD. A total of 1078 participants were included, the mean serum 25(OH)D concentration was determined to be 25 ± 18 ng/ml, with 54.45% of the participants presenting vitamin D deficiency [25(OH)D < 20 ng/mL]. Moreover, the prevalence of CVD was 59.28% in the vitamin D deficient group, which was higher than in the insufficient (48.55%) and sufficient groups (52.78%). After adjusting for potential confounders, compared with the deficient group, the ORs (95%CI) of CVDs were 0.68 (0.50, 0.91) in the insufficient group and 0.81 (0.56, 1.16) in the sufficient group. A nonlinear (U-shaped) association was observed between the risk of CVD and 25(OH)D concentration. Further research suggested that the risk of CVD was higher in males than in females. In conclusion, a U-shape association between serum levels of 25(OH)D and the risk of CVD was identified in our study, suggesting a nonlinear relationship between vitamin D with the prevalence of CVD.

## Introduction

Cardiovascular disease (CVD) is the leading cause of mortality in worldwide. Data from the World Health Organization (WHO) show that 17.7 million people died from CVD in 2015, representing 31% of all global deaths. Notably, out of all these deaths, 7.4 million people died from coronary heart disease and 6.7 million died from stroke[1]. Moreover, over three quarters of the deaths occur in developing countries[2].

As the biggest developing country, CVD is also the main cause of death in China. CVD accounted for nearly 42% of all deaths in 2010, and the economic burden of CVD was estimated to be $550 billion from 2005 to 2015[3]. Based on mortality data reported by the National Disease Surveillance Points (DSPs) system, between 2004 and 2011, the annual growth rate of CVD standardized mortality was 5.00% in men and 3.65% in women. Importantly, CVD mortality rates of rural population were higher than urban population in 2011, and continue to increase faster than the urban population[4]. In 2016, 44.8% of deaths in the rural population were due to CVD, compared to 41.9% of deaths in the urban population[5]. In this study, the CVD subcategories that we focus on including hypertension, heart failure, stroke and coronary heart disease.

Vitamin D is a fat-soluble vitamin, which signals through the vitamin D receptor (VDR) to regulate 3% to 5% of the human genome[6]. Therefore, vitamin D signals are no longer only considered to be pivotal mediators of calcium metabolism and skeletal health, but are also known to be an important regulators of cell functions, including cell differentiation and metabolism. In addition, hypovitaminosis D has been proven to be a risk factor of overall mortality[7], and vitamin D supplementation can significantly reduce the mortality[8]. Vitamin D deficiency is reported to be associated with many chronic diseases, including CVD[9–11]. Serum 25-hydroxyvitamin D (25(OH)D) is the most commonly used marker for the assessment of vitamin D nutritional status[12]. In dietary reference intakes, vitamin D deficiency is defined as a serum 25(OH)D level below 20 ng/ml, and vitamin D insufficiency as a serum 25(OH)D level of 20–29 ng/ml[13, 14]. Markedly, vitamin D deficiency has emerged as a vast public health problem, impacting almost 50% of the population around the world[15, 16]. In China, vitamin D deficiency is a larger health problem. Previous studies in Beijing and Shanghai showed that 70% of the population were vitamin D deficient, 24% had vitamin D insufficiency, only 6% of the population had sufficient levels of vitamin D[17].

In this study, a population-based survey was conducted in the rural area of Henan, China to evaluate the status of serum 25(OH)D levels, and examine the association of serum 25(OH)D concentration and CVD risk.

## Materials and methods

### Study design and population

This study was located near the 113° east longitude and; 34° north latitude. Participants aged 18 to 80 years were enrolled between July 2013 and August 2015 from three regions of the Henan province, the villages Wuzhi, Xin'an and Houzhai. The exclusion criteria were as follows: (1) recent history of acute illness; (2) treatment with drugs known to affect vitamin D metabolism in 3 months, including vitamin/ mineral supplements, because serum 25(OH)D has a half-life in the circulation of 2 weeks[18] and it can be considered to be eliminated after 5 half-life; and (3) infectious diseases, liver diseases, or advanced kidney diseases. A total of 1191 participants joined the physical examination and questionnaire of the study and 1078 participants conformed to the acceptance criteria.

### Data collection and physical measurements

After providing the written informed consent document, a standardized questionnaire designed to gather general information about the demographic characteristics, life style, medical history, and medicine use of a subject, was used in a face-to-face interview. Blood pressure was measured using an electronic sphygmomanometer (HEM-7071AFuzzy, Omron, Japan), body weight was measured using an electronic body-fat meter (V-BODY HBF-371, Omron, Japan), and electrocardiogram was measured by electrocardiogram readings (FX8600, FUKUDA, Japan), height, waist and hip circumference (without a coat or shoes) were measured to the nearest 0.1cm using a fixed measurement tape. The body mass index (BMI) of each subject was calculated using the formula: BMI = weight (kg)/ height^2^ (m^2^); the waist-to-hip ratio (WHR) was calculated using the formula: WHR = waist circumference (cm)/ hip circumference (cm); and the waist-to-height ratio (WHtR) = waist circumference (cm)/ height (cm).

### Blood sample collection

After an overnight fast, venous blood samples (about 10 mL) were collected in the morning between 6:00 to 9:00 am. Blood samples were then stored in vacuum sealed blood tubes and centrifuged under 3000 rpm for 15 minutes, and finally stored at -80°C.

### Laboratory measurements

The concentration of serum 25(OH)D was measured by an enzyme-linked immunosorbent assay (ELISA, Sangon Biotech Co. Ltd; Shanghai, China). The assay was not sensitive to two decimal places, and only integer was retained. The absorbance was measured using the microplate reader (BIO-RAD680, USA) under the wavelength of 450nm (the inter-assay CV of the total process was ≤ 9.9%). Biochemical variables, including glucose (GLU), total cholesterol (TC), triglyceride (TG), high density lipoprotein-cholesterol (HDL-C), and low density lipoprotein-cholesterol (LDL-C) were tested using an auto-biochemistry analyzer (KHB360, Shanghai, China) with the method of Hexokinase, glycerol phosphate oxidase-peroxidase (GPO-PAP), cholesterol oxidase-peroxidase (GHOD-PAP), direct method of catalase clearance and direct method of surfactant removal, respectively.

### Statistical analysis

The characteristics of the participants were expressed as the mean ± standard deviation (SD) for continuous variables and numbers (percentages) for categorical variables. The chi square (χ^2^) test was used to compare the differences of categorical variables, and one-way analysis of variance (ANOVA) / t-test (normally continuous variables) were used to compare the differences of continuous variables.

The serum 25(OH)D levels were divided into three according to the concentration, deficiency (less than 20ng/ml), insufficiency (between 20ng/ml and 30ng/ml), and sufficiency (more than 30ng/ml). The prevalence of CVD was calculated in three 25(OH)D levels and tested using the χ^2^ test. The logistic regression model was used to evaluate the odd ratios (ORs) and 95% confidence interval (95%CI) of CVD. In addition, three models were used to adjust the potential covariates. A dose-response relationship between the serum 25(OH)D and OR values of CVD was assessed using a restricted cubic spline.

Finally, a subgroup analysis was conducted to estimate the ORs of CVD in terms of the different genders, age, smoking status, drinking habits, high-fat diet, physical activity and BMI groups. The statistical analysis was conducted using the SPSS 17.0 (SPSS Inc, Chicago, IL, USA) and STATA version 11.0 (STATA Corp, College Station, Texas, USA) software package. All of the statistical analysis were two-sided and a *P*<0.05 was considered to be statistically significant.

### Ethics statement

The research was consented by the Ethics Committee of the Zhengzhou University, China. And written informed consents were signed by all participants.

## Results

### Demographic characteristics of participants

Among the 1078 participants, 428 (39.70%) were male and 650 (60.30%) were female, with a mean age of 59.60 ± 11.95 years and a mean serum 25(OH)D concentration of 25 ± 18 ng/ml. Out of all participants, 587 (54.45%) were vitamin D deficient, 311 (28.85%) were vitamin D insufficient, and 180 (16.70%) had sufficient levels of vitamin D. Overall, 594 participants were patients with CVD and the crude prevalence of CVD was 55.10%, with an age-standardized prevalence of 46.61%. Compared with non-CVD subjects, CVD patients showed significant differences in age, BMI level, blood pressure, drinking habits, physical activity, WHR, WHtR, TG and TC levels, and prevalence of type 2 diabetes (T2DM). A higher level of serum 25(OH)D was observed in non-CVDs, but was not statistically significant (Table 1).

**Table 1:** General characteristics of participants.

Variable	Non-CVD (n = 484)	CVD(n = 594)	*P*
**Male, n(%)**	202(41.74%)	226(38.05%)	0.218
**Age**	56.44 ± 11.83	62.51 ± 11.06	<0.001
**Marital status, n(%)**			0.321
Married/cohabitation	424(87.60%)	508(85.52%)	
Single/divorcement	60(12.40%)	86(14.48%)	
**Education, n(%)**			0.430
Junior high school and below	432(89.26%)	521(87.71%)	
High school and above	52(10.74%)	73(12.29%)	
**BMI(kg/m**^**2**^**)**	24.67 ± 3.43	26.18 ± 3.68	<0.001
**SBP (mm Hg)**	118.48 ± 10.62	141.85 ± 17.69	<0.001
**DBP (mm Hg)**	75.18± 7.42	85.23 ± 10.51	<0.001
**25(OH)D (ng/ml)**	25 ± 19	24 ± 18	0.203
**Smoking, n(%)**			
Never	340(70.25%)	442(74.41%)	0.128
Current/former	144(29.75%)	152(25.59%)	
**Drinking, n(%)**			0.021
Yes	91(18.80%)	81(13.64%)	
No	393(81.20%)	513(86.36%)	
**High-fat diet, n(%)**			0.091
<25g/d	383(79.13%)	494(83.16%)	
≥25g/d	101(20.87%)	100(16.84%)	
**Tea, n(%)**	62(12.81%)	75(12.63%)	0.928
**Physical activity, n(%)**			<0.001
Mild	175(36.16%)	300(50.51%)	
Moderate	81(16.73%)	101(17.00%)	
Severe	228(47.11%)	193(32.49%)	
**WHR**	0.89 ± 0.07	0.92 ± 0.07	<0.001
**Abnormal WHR, n(%)**	327(67.56%)	473(79.63%)	<0.001
**WHtR**	0.54 ± 0.06	0.57 ± 0.07	<0.001
**Abnormal WHtR, n(%)**	347(71.69%)	507(85.35%)	<0.001
**T2DM**	128(26.45%)	222(37.37%)	<0.001
**GLU(mmol/l)**	6.10 ± 2.89	6.35 ± 2.67	0.131
**TG(mmol/l)**	4.60 ± 1.01	4.75 ± 0.99	0.016
**TC(mmol/l)**	1.67 ± 1.18	1.95 ± 1.51	0.001
**HDL-C(mmol/l)**	1.23 ± 0.31	1.25 ± 0.33	0.378
**LDL-C(mmol/l)**	2.61 ± 0.80	2.68 ± 0.78	0.171

Values are mean ± standard deviation or n (%).

CVD: cardiovascular disease, BMI: body mass index, SBP: systolic blood pressure, DBP: diastolic blood pressure, WHR: waist-to-hip ratio, WHtR: waist-to-height ratio, T2DM: type 2 diabetes mellitus, GLU: glucose, TG: triglycerides, TC: total cholesterol, HDL-C: high-density lipoprotein, LDL-C: low-density lipoprotein.

### 25(OH)D concentration and prevalence of CVD

**Fig 1:**
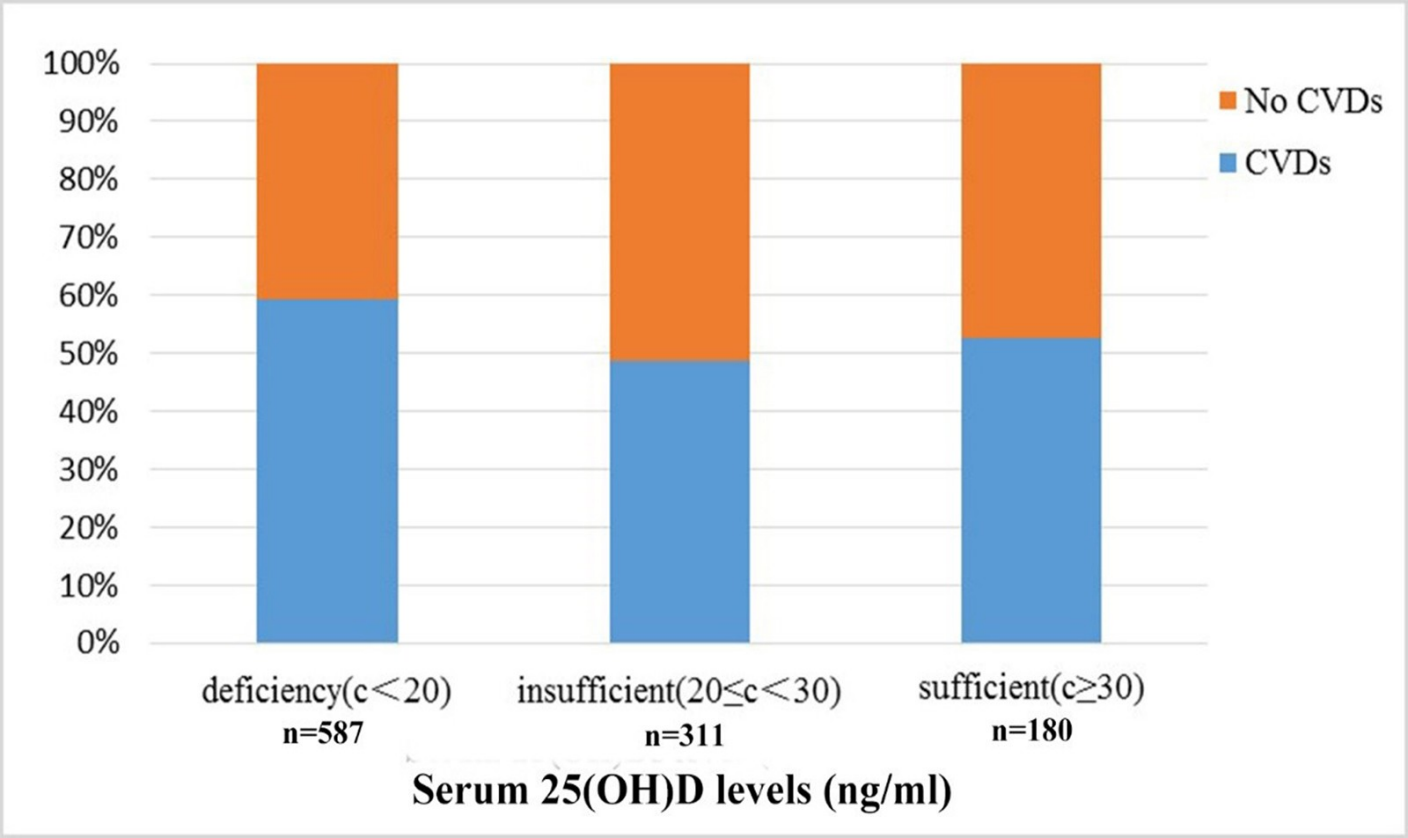
The prevalence of CVD in different serum 25(OH)D levels.

Fig 1 presents the prevalence of CVD according to the different serum 25(OH)D levels. Our results showed that the prevalence of CVD was 59.28% in the serum 25(OH)D deficient group. Moreover, a significant decrease of CVD prevalence was observed in the two other groups when compared to the serum 25(OH)D deficient group, 48.55% in the serum 25(OH)D insufficient group and 52.78% in the serum 25(OH)D sufficient group, respectively (*P* <0.001). Notably, there were no significant differences between the insufficient and sufficient groups (*P* = 0.367).

### 25(OH)D levels and risk of CVD

**Fig 2:**
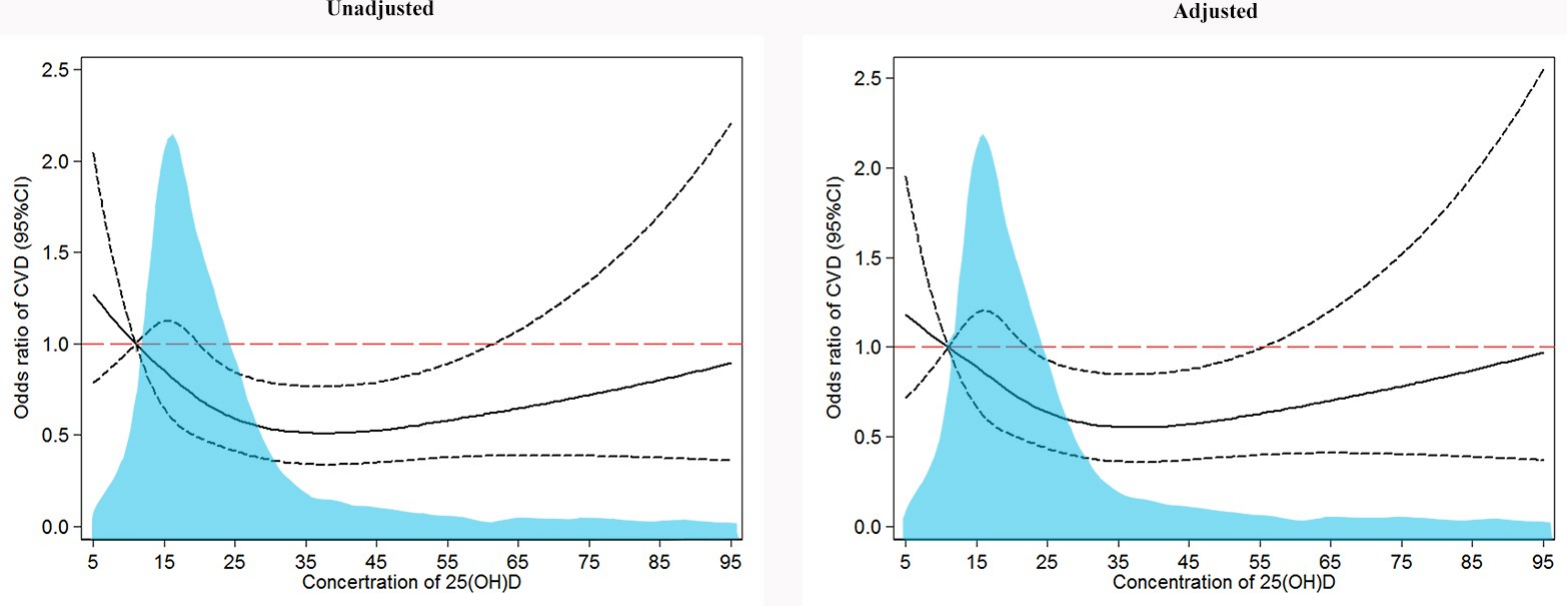
The risk of CVD according to the serum 25(OH)D concentration. Serum 25(OH)D concentration and risk of CVD in the participants. OR (solid line) and 95% CI (dashed lines) from logistic regression using restricted cubic splines. Adjusted for age, gender, education, marital status, smoking, drinking, high-fat diet, physical activity, BMI groups and T2DM (model 3). The light blue area indicates the distribution of serum 25(OH)D concentrations in participants.

As shown in Table 2, after adjustment for potential cofounders, vitamin D was critical associated with the risk of CVD, the OR (95%CI) was 1.00 (0.99, 1.00). Meanwhile, a decrease in CVD risk was observed in higher serum 25(OH)D level groups, compared with the serum 25(OH)D deficient group, the ORs (95%CI) were 0.68 (0.50, 0.91) and 0.81 (0.56, 1.16) in the insufficient and sufficient groups, respectively. In addition, spline regression analysis demonstrated a nonlinear (U-shaped) association between serum 25(OH)D concentration and risk of CVD (Fig 2). A trend χ^2^ test was also calculated to test statistical significance of the U-shaped trend (*P*_trend_ = 0.0195). The ORs of CVD decreased when the serum 25(OH)D concentration fell between 5 ng/ml and 45 ng/ml, and a uptrend resulted when the serum 25(OH)D concentration was more than 45 ng/ml.

**Table 2:** Odd ratio (95% confidence interval) for the risk of CVD according to the levels of serum 25(OH)D.

		Serum 25(OH)D levels		
Variable	Vitamin D	Deficiency	Insufficient	Sufficient
Unadjusted	1.00(0.99,1.00)	1.00	0.65(0.49,0.85)	0.77(0.55,1.07)
Model 1	1.00(0.99,1.00)	1.00	0.65(0.49,0.86)	0.80(0.57,1.14)
Model 2	1.00(0.99,1.00)	1.00	0.67(0.50,0.90)	0.80(0.56,1.14)
Model 3	1.00(0.99,1.00)	1.00	0.68(0.50,0.91)	0.81(0.56,1.16)

Model 1: Adjusted for gender, age.

Model 2: Additional adjusted education level, marital status, smoking, alcohol drinking, tea, high-fat diet, physical activity, BMI groups.

Model 3: Additional adjusted T2DM.

**Fig 3:**
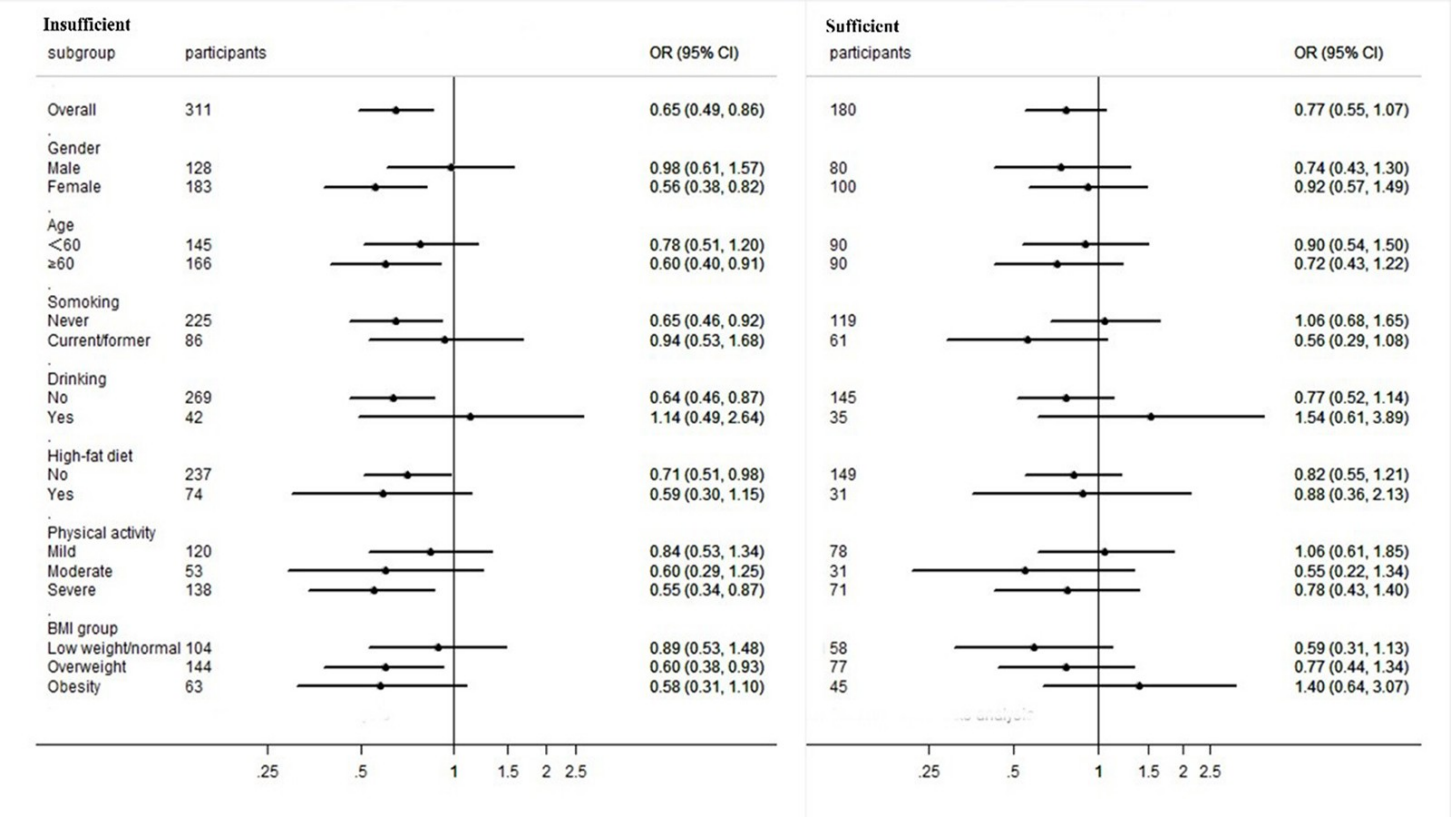
Adjusted OR (95%CI) for CVD in subgroup analysis. Subgroup analysis of serum 25(OH)D levels and risk of CVD. Circles represent the ORs, horizontal bars represent 95% CI. Adjusted for the covariates of age, gender, education, smoking, alcohol drinking, high-fat diet, physical activity, BMI groups and T2DM (model 3).

CVD is significantly associated with gender, age, smoking and drinking habits, high-fat diet, physical activity and BMI. Given this, a subgroup analysis was performed in order to assess these parameters and the results are presented in Fig 3. After full covariates adjustments (model 3), our results revealed that the ORs of CVD varied by gender, age, smoking and drinking habits, high-fat diet, physical activity and BMI of subjects in the serum 25(OH)D insufficient group. However, no significant differences were found in subjects belonging to the serum 25(OH)D sufficient group.

## Discussion

Vitamin D deficiency is a severe public health problem[17]. Many studies have suggested that vitamin D deficiency is a risk factor for T2DM, hypertension, dyslipidemia, obesity and cardiovascular disease. However, previous vitamin D studies have mainly focused on particular subjects, such as children[19], pregnant women[20] middle-aged and elderly population[17]. Therefore, our study was conducted in the rural residents of the Henan province in China, aged 18 to 80 years, covering adults of all ages. Our results indicated that 54.45% of the participants had a vitamin D deficiency, 28.85% had a vitamin D insufficiency, only 16.70% of the subjects evaluated had sufficient levels of vitamin D. Notably, the prevalence of vitamin D deficiency was more severe than what was reported in other states[21].

Furthermore, our analyses revealed that low serum 25(OH)D levels were associated with the prevalence of CVD. In the vitamin D deficiency group, the prevalence of CVD was found to be 59.28%, compared to 48.55% in the vitamin D insufficient group and 52.78% in the vitamin D sufficient group. Compared to the vitamin D deficiency group, the prevalence of CVD in the two other groups showed varying degrees of reduction. Furthermore, individuals in the insufficient group were 0.68-fold more likely to have CVD, when compared to the individuals in the vitamin D deficiency group. Meanwhile, the vitamin D sufficient group showed similar effects, although our analysis showed that they were not statistically significant. These findings suggest that vitamin D mediated signals have a protective effect on CVD, which is in line with previous studies[22–24]. Moreover, other studies have found that vitamin D are able to inhabit renin mediated gene transcription through a cAMP response element[25], which can stimulate the renin angiotensin aldosterone system (RAAS) and inhibit renin expression, leading to the down-regulation of angiotensin II[26], thereby resisting inflammatory cells and pro-inflammatory molecules within the vessel wall, such as monocyte chemoattractant protein-1, interleukin 6 and 8 (IL-6 and IL-8)[27]. In addition, vitamin D can inhibit atherosclerosis via VDRs expressed on macrophages[28] and vascular endothelium[29]. Moreover, VDRs can be expressed on the cell surface membrane and mitochondria, thus regulating multiple signaling pathways[30]. Vitamin D can then play a variety of biological effects on CVD development via VDR signals, including anti-vascular inflammation[31] and endothelial dysfunction[32].

Spline regression analysis indicated a nonlinear (U-shaped) association between serum levels of 25(OH)D and ORs of CVD. When the serum 25(OH)D concentration was less than 35 ng/ml, the ORs of CVD decreased with increasing serum 25(OH)D concentration. And there were no remarkable changes on the risk of CVD while the serum levels of 25(OH)D fluctuated between 35 ng/ml and 45 ng/ml. This illustrates that based on the state of vitamin D deficiency, raising vitamin D levels seems to be able to reduce the risk of CVD; however, based on the state of vitamin D sufficiency, additional vitamin D supplements are unable to significantly relieve the risk of CVD. Importantly, compared with vitamin insufficiency, vitamin D deficiency may have a greater impact on the development of CVD. Other studies have also reported a dose-response relationship between vitamin D and CVD. For instance, Kunutsor *et al*.,[33] found that as serum vitamin D concentrations increased by 10 ng/ml, the risk of hypertension in the population decreased by more than 10%. Anas *et al*.,[34] also demonstrated that there were dose-responses in serum 25(OH)D concentrations on arterial stiffness, with vitamin D supplementation being able to improve arterial stiffness. However, the ORs of CVD slowly increased when the serum 25(OH)D concentration was more than 45 ng/ml, although it was still less than 1. Similar results have been observed in previous study[35]. Presumably, there are important reasons for this, first, 30 to 60 ng/ml is considered to be the recommended 25(OH)D concentration[13, 36], a high concentration of serum 25(OH)D was also shown to have a protective effect on CVD; however this was not an optimal physiological concentration of 25(OH)D, therefore the ORs were slightly increased[37]. Second, there were fewer individuals with high serum 25(OH)D concentrations, and the results were not statistically significant.

In subgroup analysis, we found that the ORs varied between the different subgroups. For instance, in the serum 25(OH)D insufficient group, the ORs increased in smoking, drinking, and mild physical activity individuals, which means that these risk factors have a great impact on CVD. We were surprised to find a gender difference, with the OR of CVD being higher in males than in females. For this, we suggest that the following reasons may cause these differences: 1. Compared with females, high levels of inflammatory factors are observed in males, such as tumor necrosis factor-alpha (TNF-α), IL-6 and IL-8[38], which means a higher risk of inflammation in males, leading to harmful effects on cardiovascular function[39, 40]; 2. Hormone levels are gender specifics. Females have higher estrogen levels, and there is compelling evidence indicating that estrogen have cardiovascular protective effects. For instance, estrogen can scavenge free radicals[41], and it can also alter the expression of enzymes responsible for G-protein coupled estrogen receptor signals, thus accelerating the metabolism of reactive oxygen species[42]. These are not only limited to estrogen, other sex hormones, such as progesterone, can show cardiovascular protective effects, such as in vasodilation[43]. On the contrary, androgens can stimulate macrophages and up-regulate the expression of atherosclerosis-related genes which mediate important functions, such as lipoprotein metabolism, adhesion, inflammation, coagulation, and angiogenesis[44]. 3. Different features of atherosclerotic plaque morphology are observed between genders. For example, thrombotic plaque and plaque ulceration are seen more commonly in males than in females, with plaque necrotic lipid cores and hemorrhagic areas found more often in males[45].

The strength of this study is that our research is focused on rural areas in China, which account for a large percentage of the Chinese population, especially in Henan, the most populous province in China. Therefore, our population sample indicate a well representation of rural areas and our results can be further extrapolated to other areas. To the best of our knowledge, this is the first time a study focuses on the vitamin D levels and risk of CVD in the rural Chinese population. Nevertheless, there are several limitations in our study: First, there is a possibility of report bias in covariates, such as lifestyle, however, statistical analyses were adjusted for known confounding factors as much as possible. Second, there is a seasonal variation in the serum 25-hydroxyvitamin D levels, in summer, due to the sunlight and skin exposure, the serum 25-hydroxyvitamin D levels may higher, and the research was conducted in July and August of each year, so the serum 25-hydroxyvitamin D levels of participants were in a higher stage of the year, so the VD deficiency may be more severe than expected. Third, given that this study was designed as a cross-sectional study, the temporal relationship between vitamin D levels and CVD risk cannot be formally assessed.

## Conclusion

Our study suggests that lower serum 25(OH)D levels are associated with an increased risk of CVD in the Chinese rural population, with a nonlinear (U-shape) association between 25(OH)D levels and CVD risk. Nonetheless, further studies are needed to verify these findings and follow-up work should focus on the clinical implications of vitamin D signals on CVD prevention and treatment.

## Supporting information

S1 Table Minimal anonymized data. (XLSX)

S1 Text Questionnaire in Chinese. (DOCX)

S2 Text Questionnaire in English. (DOCX)
