# Supplementary material for: Association between vitamin D and risk of cardiovascular disease in Chinese rural population
Source: PLoS One. 2019 May 23;14(5):e0217311. doi: 10.1371/journal.pone.0217311 (PMC6532968; doi:10.1371/journal.pone.0217311)
Supplement: S1 Text — (DOCX) [file pone.0217311.s002.docx]

**慢性非传染性疾病及健康影响因素调查表**

| **1.** 户主姓名： **2.** 被调查者姓名： |
| --- |
| **3.** 身份证号码：□□□□□□□□□□□□□□□□□□ |
| **4.** 家庭住址：   市 区(县)   镇（乡） 街道(村) 队/组 号 |
| **5.** 电话： （家） (手机) |
| **6.** 联系人姓名： 关系： 电话(与本人不同)： |
| **7.** 顺序编码(ID):□ □ □ □ □ □  **8.** 调查地编码: □ □ |
| **9.** 调查日期： 年 月 日 |
| **10.** 调查开始时间：  时 分 |

| **一、一般情况** | | | | | | | | | |
| --- | --- | --- | --- | --- | --- | --- | --- | --- | --- |
| **A1.** 居住地：1=城市 2=农村 □ | | | | | **A2.** 性别：1=男性 2=女性 □ | | | | |
| **A3.** 民族：1=汉 8=其它 □ | | | | | **A4.** 宗教信仰：0=无 1=有 □ | | | | |
| **A5.** 出生日期： 年 月 日 1=阳历 2=阴历 □ | | | | | | | | | |
| **A6.** 文化程度： □  1=未上学 2=小学 3=初中 4=高中/中专 5=大专/大学 6=硕士及以上 | | | | | | | | | |
| **A7.** 婚姻状况： 1=结婚/同居 2=丧偶 3=离婚/分居 4=未婚 □ | | | | | | | | | |
| **A8.** 职业： □  1=工人(包括农民工) 2=农民    3=管理人员/干部   4=专业技术人员  5=商业服务人员 6=个体经营者 7=退休/离休   8=家务  9=其它（注明： ） | | | | | | | | | |
| **A9.**家庭人口数 ，去年您全家总收入为人民币 元？ | | | | | | | | | |
| **A10.**您的家庭人均月收入为以下哪个水平？ □  1=500元以下 2=500~999元 3=1000~1999元 4=2000~2999元 5=3000元及以上 | | | | | | | | | |
| **A11.** 您享受下列哪种医疗服务？（请选主要的一项） □  1=新农合   2=城镇职工医疗保险 3=城镇居民医疗保险 4=商业医疗保险  5=公费医疗 8=其它 | | | | | | | | | |
| **二、生活习惯** | | | | | | | | | |
| **（一）吸烟情况** | | | | | | | | | |
| **B1.** 您是否吸烟（吸烟指每天至少吸一支烟并持续**半年**以上） □ | | | | | | | | | |
| 0=从不吸烟或仅偶尔吸少量烟（平均每天不超过一支）（*转问****B2***） | | | | | | | | | |
| 1=现在吸烟 | **B1a.** 开始吸烟：_____岁  **B1b.** 现在平均每天吸：_______支 （*转问****B3***） | | | | | | | | |
| 2=现已戒烟 | **B1c.** 开始吸烟：_____岁  **B1d.** 戒烟前平均每天吸：_____支  **B1e.** 戒烟时年龄：_____岁  **B1f.** 您戒烟的主要原因是： □  1=经济原因 2= 健康原因  3=家人/朋友反对 8=其它（注明： ）（*转问****B3***） | | | | | | | | |
| **B2.** 您有没有吸入别人吸烟产生的烟雾（被动吸烟：不吸烟者一天内吸入吸烟者呼出的烟雾至少15分钟）？ | | | | | | | | | |
| 0=没有或基本没有（*转问****B3***） | | | | | | | | | |
| 1=有 | **B2a.** 您平均每周被动吸烟 天数（9=不详） | | | | | | | | |
|  | **B2b.** 您平均每天被动吸烟 小时 分钟（99=不详） | | | | | | | | |
|  | **B2c.** 您被动吸烟了 年 | | | | | | | | |
| **（二）饮酒情况** | | | | | | | | | |
| **B3.** 您是否饮酒（**每年**至少饮酒**12**次以上）？ □ | | | | | | | | | |
| 0=从不饮酒或仅偶尔饮少量酒（*转问****B7***） | | | | | | | | | |
| 1=现在饮酒 | | | **B3a**. 开始饮酒年龄： 岁 （*转问****B4***） | | | | | | |
| 2=现已戒酒 | | | **B3b.** 开始饮酒年龄： 岁  **B3c.** 戒酒时的年龄： 岁  **B3d.** 您戒酒的主要原因是： □  1=经济原因 2=健康原因  3=家人/朋友反对 8=其它（注明： ） | | | | | | |
| **B4.** 如果饮酒属于哪一种？（包括***现在饮酒***和***现已戒酒***） □  1=每天至少饮酒1次 2=每周至少饮酒1次 3=每月至少饮酒1次 | | | | | | | | | |
| **B5.** 饮酒的种类、频率和饮酒量（包括***现在饮酒***和***现已戒酒***的情况） | | | | | | | | | |
| **种类** | | **饮用频率（选择其中一种）** | | | | **次数** | **每次饮酒量** | | **月/年** |
| **B5a.** 啤酒 | | 0=不饮  1=天  2=周  3=月  4=年 | | | |  | 瓶/次 | |  |
| **B5b.**白酒 | | 0=不饮  1=天  2=周  3=月  4=年 | | | |  | 两/次 | |  |
| **B5c.**葡萄酒/红酒 | | 0=不饮  1=天  2=周  3=月  4=年 | | | |  | 两/次 | |  |
| **B5d.**黄酒/米酒 | | 0=不饮  1=天  2=周  3=月  4=年 | | | |  | 两/次 | |  |
| **B6.** 您饮酒是否经常喝醉？（包括***现在饮酒***和***现已戒酒***） □  1=几乎每次 2=绝大多数 3=偶尔 4=从不 | | | | | | | | | |
| **食物摄取（记录过去一年您摄入下列食物的频率和食用量）** | | | | | | | | | |
| **食物种类** | | | | **食用频度（选择其中一种）** | | | | **食用量** | |
| **B7.**主食 | | | | 0=不吃  1=天  2=周  3=月  4=年 □ | | | | 斤 两 | |
| **B8.**猪牛羊肉 | | | | 0=不吃  1=天  2=周  3=月  4=年 □ | | | | 斤 两 | |
| **B9.**鸡、鸭肉 | | | | 0=不吃  1=天  2=周  3=月  4=年 □ | | | | 斤 两 | |
| **B10.**各种鱼类 | | | | 0=不吃  1=天  2=周  3=月  4=年 □ | | | | 斤 两 | |
| **B11.**鸡蛋、鸭蛋等蛋类 | | | | 0=不吃  1=天  2=周  3=月  4=年 □ | | | | 个 | |
| **B12.**牛奶、酸奶等奶制品 | | | | 0=不吃  1=天  2=周  3=月  4=年 □ | | | | ml | |
| **B13.**新鲜水果 | | | | 0=不吃  1=天  2=周  3=月  4=年 □ | | | | 斤 两 | |
| **B14.**新鲜蔬菜 | | | | 0=不吃  1=天  2=周  3=月  4=年 □ | | | | 斤 两 | |
| **B15.**豆类及豆制品 | | | | 0=不吃  1=天  2=周  3=月  4=年 □ | | | | 斤 两 | |
| **B16.**干果（瓜子、花生等） | | | | 0=不吃  1=天  2=周  3=月  4=年 □ | | | | 斤 两 | |
| **B17.**咸菜/腌菜 | | | | 0=不吃  1=天  2=周  3=月  4=年 □ | | | | 斤 两 | |
| **B18.**杂粮（玉米、薯类、高粱等） | | | | 0=不吃  1=天  2=周  3=月  4=年 □ | | | | 斤 两 | |
| **B19.**动物油(猪/牛油等) | | | | 0=不吃  1=天  2=周  3=月  4=年 □ | | | | 斤 两 | |
| **B20.**您是否经常喝茶？（加茶叶，经常指**每周至少1次**，持续喝茶6个月以上）  0=否（*转问****C1***） 1=是 □ | | | | | | | | | |
| **B21.**您最常喝哪种茶？ □  1=绿茶 2=红茶（普洱、乌龙茶等）  3=花茶（菊花茶、玫瑰花茶等） 8=其它 | | | | | | | | | |
| **B22.**您平均**每周**有 天喝这种茶。 | | | | | | | | | |
| **（三）体力活动（询问最近7天的体力活动状况）** | | | | | | | | | |
| *请回忆您****最近7天****所做的****剧烈体力活动****（指需要费很大力气并使您气喘吁吁的活动）。只考虑每次持续****10分钟****以上的活动。*  **C1. 最近7天**中您是否进行过**剧烈体力活动**（如锄地、挖地等重体力耕作、建筑和装修、人力搬运、采矿、炼钢等；快跑、快走、快速骑车、登山等）？ □  0=否，无剧烈活动（*转问****C3***） 1=是， 天/周  **C2.** 有**剧烈体力活动**的日子里，您通常每天活动多长时间？ □  0=不清楚 1=是， 小时 分钟/天 | | | | | | | | | |
| *请回忆您****最近7天****所做的****适度体力活动****（指需要适度力气并使您呼吸较平常稍加快的活动）。只考虑每次****持续10分钟****以上的活动。*  **C3. 最近7天**中您是否进行了**适度体力活动**（如家务、带孩子、整理庭院、驾车、电工、木工、教学等；慢跑、跳舞、秧歌、健身操、一般速度骑车等，但不包括走路）？ □  0=否，无适度体力活动（*转问****C5***） 1=是， 天/周  **C4.** 有**适度体力活动**的日子里，您通常每天活动多长时间？ □  0=不清楚 1=是， 小时 分钟/天 | | | | | | | | | |
| *请回忆您****最近7天****花在****走路活动****上的时间（包括您下地和在家中的走动，还包括下地路途以及您为了锻炼、消遣和休闲时的走路/散步）。*  **C5. 最近7天**中您是否进行过**每次10分钟**以上的**走路活动**？ □  0=否，没有走路（*转问****C7***） 1=是， 天/周  **C6.** 有**走路活动**的日子里，您通常每天**走动**多长时间？ □  0=不清楚 1=是， 小时 分钟/天 | | | | | | | | | |
| *请回忆您****最近7天****有多长时间是****坐着****的（包括下地/上班和在家时坐着的总时间，如看书、看电视/电脑/手机、吃饭、聊天、玩棋牌等）。*  **C7. 最近7天**中，您通常每天有多长时间处于**坐位**？ □  0=不清楚 1=是， 小时 分钟/天  其中坐着**看电视/影碟/电脑/手机**  小时 分钟/天 | | | | | | | | | |

| **四、疾病史、用药史与家族疾病史**（根据**医生/医院确诊**结果填写） | | | | | | | | | | |
| --- | --- | --- | --- | --- | --- | --- | --- | --- | --- | --- |
| **疾病名称** | **个人疾病史** | **药物类型** | **您是否使用过上述药物**  （使用时间） | **最近两周是否使用过上述药物** | **直系亲属患病史（可多选）** | | | | | |
|  |  |  |  |  | **父亲** | **母亲** | **兄弟**  **姐妹** | **子女** | **不详** | **无** |
| **E1.** 高血压 | 0=无 1=有 岁确诊 | 降压药 | 0=否 1=是（ 年 月） | 0=否 1=是 | □ | □ | □ | □ | □ | □ |
| **E2.** 高脂血症 | 0=无 1=有 岁确诊 | 降脂药 | 0=否 1=是（ 年 月） | 0=否 1=是 | □ | □ | □ | □ | □ | □ |
| **E3.** 糖尿病 | 0=无 1=有 岁确诊 | 口服降糖药 | 0=否 1=是（ 年 月） | 0=否 1=是 | □ | □ | □ | □ | □ | □ |
|  |  | 胰岛素 | 0=否 1=是（ 年 月） | 0=否 1=是 |  |  |  |  |  |  |
| **E4.** 冠心病 | 0=无 1=有 岁确诊 | 治疗性药物 | 0=否 1=是（ 年 月） | 0=否 1=是 | □ | □ | □ | □ | □ | □ |
| **E4a.** 如果有 | （可多选）：1=心肌梗死 2=心绞痛 3=心律失常 4=心衰 8=其它 9=不详 □ | | | | | | | | | |
| **E5.** 脑卒中 | 0=无 1=有 岁确诊 | 治疗性药物 | 0=否 1=是（ 年 月） | 0=否 1=是 | □ | □ | □ | □ | □ | □ |
| **E5a.** 如果有 | （可多选）：1=脑梗塞 2=脑出血 8=其它 9=不详 □ | | | | | | | | | |
| **E6.** 肺气肿 | 0=无 1=有 岁确诊 | 治疗性药物 | 0=否 1=是（ 年 月） | 0=否 1=是 | □ | □ | □ | □ | □ | □ |
| **E7.** 慢性支气管炎 | 0=无 1=有 岁确诊 | 治疗性药物 | 0=否 1=是（ 年 月） | 0=否 1=是 | □ | □ | □ | □ | □ | □ |
| **E8.** 哮喘 | 0=无 1=有 岁确诊 | 治疗性药物 | 0=否 1=是（ 年 月） | 0=否 1=是 | □ | □ | □ | □ | □ | □ |
| **E9.** 慢阻肺/COPD | 0=无 1=有 岁确诊 | 治疗性药物 | 0=否 1=是（ 年 月） | 0=否 1=是 | □ | □ | □ | □ | □ | □ |
| **E10.** 过敏性鼻炎 | 0=无 1=有 岁确诊 | 治疗性药物 | 0=否 1=是（ 年 月） | 0=否 1=是 | □ | □ | □ | □ | □ | □ |
| **E11.** 肿瘤 | 0=无 1=有 岁确诊 | 抗肿瘤药物 | 0=否 1=是（ 年 月） | 0=否 1=是 | □ | □ | □ | □ | □ | □ |
| **E11a.** 如果有 | 请填写名称： 、 、 | | | | | | | | | |
| **E12.** 肾脏疾病 | 0=无 1=有 岁确诊 | 治疗性药物 | 0=否 1=是（ 年 ） | 0=否 1=是 | □ | □ | □ | □ | □ | □ |
| **E12a.** 如果有 | （可多选）：1=肾结石 2=肾炎 3=肾囊肿 4=肾衰竭 5=输尿管结石 8=其它 9=不详 □ | | | | | | | | | |
| **E13.** 肝脏疾病 | 0=无 1=有 岁确诊 | 治疗性药物 | 0=否 1=是（ 年 ） | 0=否 1=是 | □ | □ | □ | □ | □ | □ |
| **E13a.** 如果有 | （可多选）：1=脂肪肝 2=肝硬化 3=肝囊肿 4=肝脓肿 8=其它 9=不详 □ | | | | | | | | | |
| **E14.** 慢性肝炎 | 0=无 1=有 岁确诊 | 治疗性药物 | 0=否 1=是（ 年 ） | 0=否 1=是 | □ | □ | □ | □ | □ | □ |
| **E14a.** 如果有 | （可多选）：1=病毒性肝炎 2=酒精性肝炎 3=药物性肝炎 8=其它 9=不详 □ | | | | | | | | | |
| **E15.** 胆囊疾病 | 0=无 1=有 岁确诊 | 治疗性药物 | 0=否 1=是（ 年 ） | 0=否 1=是 | □ | □ | □ | □ | □ | □ |
| **E15a.** 如果有 | （可多选）：1=胆结石 2=胆囊炎 8=其它 9=不详 □ | | | | | | | | | |
| **E16.** 胰腺疾病 | 0=无 1=有 岁确诊 | 治疗性药物 | 0=否 1=是（ 年 ） | 0=否 1=是 | □ | □ | □ | □ | □ | □ |
| **E16a.** 如果有 | （可多选）：1=急性胰腺炎 2=慢性胰腺炎 8=其它 9=不详 □ | | | | | | | | | |
| **E17.** 胃肠疾病 | 0=无 1=有 岁确诊 | 治疗性药物 | 0=否 1=是（ 年 ） | 0=否 1=是 | □ | □ | □ | □ | □ | □ |
| **E17a.** 如果有 | （可多选）：1=胃溃疡 2=十二指肠溃疡 3=胃炎 4=慢性结肠炎 5=胃食管反流病 6=便秘 8=其它 9=不详 □ | | | | | | | | | |
| **E18.** 皮肤疾病 | 0=无 1=有 岁确诊 | 治疗性药物 | 0=否 1=是（ 年 ） | 0=否 1=是 | □ | □ | □ | □ | □ | □ |
| **E18a.** 如果有 | （可多选）：1=湿疹 2=接触性皮炎 3=荨麻疹 4=银屑病 5=白癜风 8=其它 9=不详 □ | | | | | | | | | |
| **E19.** 痛风 | 0=无 1=有 岁确诊 | 治疗性药物 | 0=否 1=是（ 年 ） | 0=否 1=是 | □ | □ | □ | □ | □ | □ |
| **E20.** 肺结核 | 0=无 1=有 岁确诊 | 治疗性药物 | 0=否 1=是（ 年 ） | 0=否 1=是 | □ | □ | □ | □ | □ | □ |
| **E21.** **最近一年**，您看病/就诊次数为 次; 住院次数为 次. | | | | | | | | | | |
| **E22. 最近两周**，您的身体是否有病伤的情况？ 0=否（*转问****E23***） 1=是 □ | | | | | | | | | | |
| **E22a.** 1=没有就诊，也没有自服药物或采取辅助疗法 2=没有就诊，但自服了药物或采取了一些辅助疗法 3=去医疗卫生单位就诊 □ | | | | | | | | | | |
| **E23.** 最近两周，是否服用过什么药物？（可多选） 0=否 1=是 | | | | | | | | | | |
| 1=止痛药 2=安眠药 3=止咳药 4=利尿剂 5=治疗哮喘药 6=溶栓剂 7=补钙药 8=抗生素 9=激素类药 10=抗凝血药  11=维生素 （1 复合维生素 2 维生素D 3 其他 ） 12=阿司匹林类（使用 年 月） 13=其他 | | | | | | | | | | |

| **五、身体功能（请选择最适合您的选项）** |
| --- |
| **E1.** 今天您在行动（四处走动）方面： □  1=没有困难 2=有一点困难 3=有中度困难 4=有严重困难 5=无法四处走动 |
| **E2.** 今天您在自我照顾（洗脸、刷牙、穿衣、上厕所等）方面： □  1=没有困难 2=有一点困难 3=有中度困难 4=有严重困难 5=无法自我照顾 |
| **E3.** 今天您从事日常活动（工作、读书或做家务）方面： □  1=没有困难 2=有一点困难 3=有中度困难 4=有严重困难 5=无法进行日常活动 |
| **E4.** 今天您身体疼痛或不舒服方面： □  1=无 2=有一点 3=中度 4=严重 5=极度 |
| **E5.** 今天您在焦虑（如紧张、担心、不安等）和/或抑郁（如做事情缺乏兴趣、提不起精神等）方面： □  1=无 2=有一点 3=中度 4=严重 5=极度 |
| **E6.** 如果您给自己今天的健康状况打一个总分，您打 分？  （满分为100分，分值越高，越健康）  ├---┼—-┼—-┼—-┼—-┼—-┼—-┼—-┼—-┼—-┤  0 10 20 30 40 50 60 70 80 90 100 |
| 调查者签名： 调查结束时间： 时 分 |
| 请您对本次调查的质量进行评价： 1=高 2=中等 3=低 □ |

| **体 格 检 查 指 标** |
| --- |
| **第一次测量 第二次测量**  **F1.** 身高   厘米 厘米（精确到0.1厘米）  **F2.** 腰围   厘米 厘米（精确到0.1厘米）  **F3.** 臀围   厘米 厘米（精确到0.1厘米）  检查者签名： |
| **F4.** 体 重  公斤（精确到0.1公斤）  **F5.** 身体脂肪率 %（精确到0.1%）  **F6.** 基础代谢 千卡  **F7.** 内脏脂肪指数  检查者签名： |
| **左手 / 右手**  **F8.** 第1次测量读数  / 公斤（精确到0.1公斤）  **F9.** 第2次测量读数  / 公斤（精确到0.1公斤）  **F10.** 第3次测量读数  / 公斤（精确到0.1公斤）  **F11.** 优势手 1=左手 2=右手 □  检查者签名： |
| **F12.** 静坐休息5分钟，测量血压和脉搏**（两次测量之间休息不低于30秒）：**  **静息脉搏**  第1次血压读数 / mmHg  次/分钟  第2次血压读数 / mmHg  次/分钟  第3次血压读数 / mmHg  次/分钟  检查者签名： |
