# Supplementary material for: Association between vitamin D and risk of cardiovascular disease in Chinese rural population
Source: PLoS One. 2019 May 23;14(5):e0217311. doi: 10.1371/journal.pone.0217311 (PMC6532968; doi:10.1371/journal.pone.0217311)
Supplement: S2 Text — (DOCX) [file pone.0217311.s003.docx]

**Questionnaire on CND and health impact factors**

| **1.** Family Name： **2.** Name： |
| --- |
| **3.** National ID number：□□□□□□□□□□□□□□□□□□ |
| **4.** Home address：______ Province ______ City________ District/County ______ Street/Village |
| **5.** Telephone： |
| **6.** Contact name： relation： Telephone： |
| **7.** ID:□ □ □ □ □ □  **8.** Village ID: □ □ |
| **9.** Data of survey： year month day |
| **10.** Time of survey：  clock min |

| **A、General information** | | | | | | | | | |
| --- | --- | --- | --- | --- | --- | --- | --- | --- | --- |
| **A1.** Inhabitation:1=urban 2=rural □ | | | | | **A2.** Gender：1=male 2=female □ | | | | |
| **A3.** Nation：1=Han 8=Other □ | | | | | **A4.** Religion：0=No 1=Yes □ | | | | |
| **A5.** Date of birth： year month day 1= solar calendar 2= lunar calendar □ | | | | | | | | | |
| **A6.** Education： □  1= No formal school 2= Primary School 3= Middle School 4= High School 5= Technical school / college 6= University | | | | | | | | | |
| **A7.** Marital status： 1= Married/cohabitation 2= widowed 3= divorcement 4=single □ | | | | | | | | | |
| **A8.** Occupation： □  1= Factory worker 2= Agriculture & related workers 3= Administrator / manager  4= Professional / technical 5= Sales & service workers 6= Self-employed  7= Retired   8= House wife / husband  9= Other（stated： ） | | | | | | | | | |
| **A9.**Family number ，income | | | | | | | | | |
| **A10.**income per head？ □  1=500 and below 2=500~999 3=1000~1999 4=2000~2999 5=3000 and above | | | | | | | | | |
| **A11.** Which kind of medical service do you have？ □  1= New rural cooperative medical care   2= Medical insurance for urban workers  3= Medical insurance for urban resident 4= Commercial health insurance  5= Socialized medicine 8=Other | | | | | | | | | |
| **B、Habit** | | | | | | | | | |
| **（1）Smoking** | | | | | | | | | |
| **B1.** Did you smoke? □ | | | | | | | | | |
| 0=Never or less than once a day （*go to* ***B2***） | | | | | | | | | |
| 1=Yes | **B1a.** When does it start：_____year  **B1b.** How many cigarettes a day? _______ （*go to* ***B3***） | | | | | | | | |
| 2=Quitted | **B1c.** When does it start：_____year  **B1d.** How many cigarettes a day? _____  **B1e.** Age when quitted smoking：_____  **B1f.** The main reason for quitted smoking: □  1= Economic 2= Health  3= Family / friend objection  8=Other（Stated： ）（*go to* ***B3***） | | | | | | | | |
| **B2.** Passive smoking？ | | | | | | | | | |
| 0=No（*go to* ***B3***） | | | | | | | | | |
| 1=Yes | **B2a.** How many days a week? day（9=unknown） | | | | | | | | |
|  | **B2b.** How long each day hour min（99=unknown） | | | | | | | | |
|  | **B2c.** How many years? year | | | | | | | | |
| **（2）Drinking** | | | | | | | | | |
| **B3.** Did you drink?（more than 12 times a year）？ □ | | | | | | | | | |
| 0=Never or little（*go to* ***B7***） | | | | | | | | | |
| 1=Drink now | | | **B3a**. When dose it start? year （*go to* ***B4***） | | | | | | |
| 2=Quitted | | | **B3b.** When dose it start? year  **B3c.** Age when quitted drinking： year  **B3d.** The main reason for quitted drinking： □  1=Economic 2=Health  3= Family / friend objection  8=Other（Stated： ） | | | | | | |
| **B4.** How frequently do you drink？ □  1=at least once a day 2=at least once a week 3=at least once a month | | | | | | | | | |
| **B5.** Type, frequency and amount of alcohol consumed（包括***现在饮酒***和***现已戒酒***的情况） | | | | | | | | | |
| **Type** | | **Frequency** | | | | **Time** | **Amount** | | **month/year** |
| **B5a.** Beer | | 0=No  1=day  2=week  3=month  4=year | | | |  | 瓶/次 | |  |
| **B5b.**Spirit | | 0=No  1=day  2=week  3=month  4=year | | | |  | 两/次 | |  |
| **B5c.**Wine | | 0=No  1=day  2=week  3=month  4=year | | | |  | 两/次 | |  |
| **B5d.**Rice wine | | 0=No  1=day  2=week  3=month  4=year | | | |  | 两/次 | |  |
| **B6.** Do you often drunk？ □  1=everytime 2=always 3=sometimes 4=never | | | | | | | | | |
| **(3) Food consumption** | | | | | | | | | |
| **Kind** | | | | **Frequency** | | | | **Amount** | |
| **B7.**Staple food | | | | 0=never 1=day 2=week 3=month 4=year □ | | | | gram | |
| **B8.**Animal meat | | | | 0=never 1=day 2=week 3=month 4=year □ | | | | gram | |
| **B9.**Poultry | | | | 0=never 1=day 2=week 3=month 4=year □ | | | | gram | |
| **B10.**Fish | | | | 0=never 1=day 2=week 3=month 4=year □ | | | | gram | |
| **B11.**Eggs, duck eggs, etc | | | | 0=never 1=day 2=week 3=month 4=year □ | | | |  | |
| **B12.**Milk, yogurt, etc | | | | 0=never 1=day 2=week 3=month 4=year □ | | | | ml | |
| **B13.**Fruit | | | | 0=never 1=day 2=week 3=month 4=year □ | | | | gram | |
| **B14.**Vegetable | | | | 0=never 1=day 2=week 3=month 4=year □ | | | | gram | |
| **B15.**beans and products | | | | 0=never 1=day 2=week 3=month 4=year □ | | | | gram | |
| **B16.**Dry fruit | | | | 0=never 1=day 2=week 3=month 4=year □ | | | | gram | |
| **B17.**Pickles | | | | 0=never 1=day 2=week 3=month 4=year □ | | | | gram | |
| **B18.**Roughage | | | | 0=never 1=day 2=week 3=month 4=year □ | | | | gram | |
| **B19.**Animal oil | | | | 0=never 1=day 2=week 3=month 4=year □ | | | | gram | |
| **B20.**Do you often have tea?（at least once a week for more than 6 months）  0=No（*go to* ***C1***） 1=Yes □ | | | | | | | | | |
| **B21.**What kind of tea do you drink most often? □  1=Green tea 2=Red tea  3= Scented tea（Chrysanthemum, rose, etc） 8=Other | | | | | | | | | |
| **B22.**How many days a week do you drink this tea? day | | | | | | | | | |
| **C、Body exercise（in last 7 days）** | | | | | | | | | |
| *Intense physical activity during the last 7 days (only activities that last more than 10 minutes).*  **C1.** Have you had intense physical activity in the last 7 days (such as hoeing, digging, farming, construction and decoration, manual handling, mining, steelmaking, running, walking, cycling, climbing, etc.)? □  0=No（*go to* ***C3***） 1=Yes， day/week  **C2.** How long do you usually have intense physical activity every day? □  0=Unknown 1=Yes， hour min/day | | | | | | | | | |
| *Medium physical activity during the last 7 days (only activities that last more than 10 minutes).*  **C3.** Do you have moderate physical activity in the last 7 days (such as housework, babysitting, clean courtyard, driving, electricians, carpentry, teaching, jogging, dancing, body-building, general speed cycling, etc., but excluding walking)? □  0=No（*go to* ***C5***） 1=Yes， day/week  **C4.** How long do you usually have moderate physical activity every day? □  0=Unknown 1=Yes， hour min/day | | | | | | | | | |
| *How much time you have spent on walking in the last 7 days (including trip to the ground and home, as well as walk for exercise, recreation and leisure).*  **C5.** Have you had more than 10 minutes of walking in the last 7 days? □  0=No（*go to* ***C7***） 1= Yes， day/week  **C6.** How long do you usually walk ? □  0=Unknown 1=Yes， hour min/day | | | | | | | | | |
| *How long have you been sitting in the last 7 days* *(including the total amount of time spent sitting at work and home, such as reading, watching TV / computer / cell phone, eating, chatting, playing chess cards, etc.).*  **C7.** How long have you been sitting in each day? □  0=Unknown 1=Yes， hour min/day  Time spent on watch/computer/phone hour min/day | | | | | | | | | |

| **D、History of disease/** **medication/ family disease (based on results of doctor / hospital diagnosis)** | | | | | | | | | | |
| --- | --- | --- | --- | --- | --- | --- | --- | --- | --- | --- |
| **Disease** | **No/ Yes(age at first diagnosis)** | **Medication** | **Do you use the drugs (use time)** | **Have you used these drugs in the last two weeks?** | **History of immediate family disease (****optional)** | | | | | |
|  |  |  |  |  | **Father** | **Mother** | **Siblings** | **Children** | **Unknown** | **None** |
| **D1.** Hypertension | 0=No 1=Yes year | Hypotensor | 0=No 1=Yes（ year month） | 0=No 1=Yes | □ | □ | □ | □ | □ | □ |
| **D2.** Hyperlipemia | 0=No 1=Yes year | Antilipemic | 0=No 1=Yes（ year month） | 0=No 1=Yes | □ | □ | □ | □ | □ | □ |
| **D3.** Diabete | 0=No 1=Yes year | hypoglycemic agents | 0=No 1=Yes（ year month） | 0=No 1=Yes | □ | □ | □ | □ | □ | □ |
|  |  | Insulin | 0=No 1=Yes（ year month） | 0=No 1=Yes |  |  |  |  |  |  |
| **D4.**Coronary disease | 0=No 1=Yes year | Treatment | 0=No 1=Yes（ year month） | 0=No 1=Yes | □ | □ | □ | □ | □ | □ |
| **D4a.** If yes | （optional）：1= myocardial infarction 2= angina pectoris 3= arrhythmia 4= heart failure 8=other 9=unknown □ | | | | | | | | | |
| **D5.** Stroke | 0=No 1=Yes year | Treatment | 0=No 1=Yes（ year month） | 0=No 1=Yes | □ | □ | □ | □ | □ | □ |
| **D5a.** If yes | （optional）：1=erebral infarction 2= cerebral hemorrhage 8=other 9=unknown □ | | | | | | | | | |
| **D6.** Pneumonectasis | 0=No 1=Yes year | Treatment | 0=No 1=Yes（ year month） | 0=No 1=Yes | □ | □ | □ | □ | □ | □ |
| **D7.**Chronic bronchitis | 0=No 1=Yes year | Treatment | 0=No 1=Yes（ year month） | 0=No 1=Yes | □ | □ | □ | □ | □ | □ |
| **D8.** Asthma | 0=No 1=Yes year | Treatment | 0=No 1=Yes（ year month） | 0=No 1=Yes | □ | □ | □ | □ | □ | □ |
| **D9.** COPD | 0=No 1=Yes year | Treatment | 0=No 1=Yes（ year month） | 0=No 1=Yes | □ | □ | □ | □ | □ | □ |
| **D10.** Rhinallergosis | 0=No 1=Yes year | Treatment | 0=No 1=Yes（ year month） | 0=No 1=Yes | □ | □ | □ | □ | □ | □ |
| **D11.** Cancer | 0=No 1=Yes year | Antitumor treatment | 0=No 1=Yes（ year month） | 0=No 1=Yes | □ | □ | □ | □ | □ | □ |
| **D11a.** If yes | What tumor： 、 、 | | | | | | | | | |
| **D12.**Nephropathy | 0=No 1=Yes year | Treatment | 0=No 1=Yes（ year month） | 0=No 1=Yes | □ | □ | □ | □ | □ | □ |
| **D12a.** If yes | （Optional）：1= nephrolithiasis 2= nephritis 3= renal cyst 4= renal failure 5= ureteral stone 8=other  9=unknown □ | | | | | | | | | |
| **D13.** Hepatic disease | 0=No 1=Yes year | Treatment | 0=No 1=Yes（ year month） | 0=No 1=Yes | □ | □ | □ | □ | □ | □ |
| **D13a.** If yes | （Optional）：1= fatty liver 2= cirrhosis 3= liver cyst 4= liver abscess 8=other 9=unknown □ | | | | | | | | | |
| **D14.** Chronic hepatitis | 0=No 1=Yes year | Treatment | 0=No 1=Yes（ year month） | 0=No 1=Yes | □ | □ | □ | □ | □ | □ |
| **D14a.** If yes | （Optional）：1= viral hepatitis 2= alcoholic hepatitis 3= drug-induced hepatitis 8=other 9=unknown □ | | | | | | | | | |
| **D15.** Gallbladder disease | 0=No 1=Yes year | Treatment | 0=No 1=Yes（ year month） | 0=No 1=Yes | □ | □ | □ | □ | □ | □ |
| **D15a.** If yes | （Optional）：1= gallstone 2= cholecystitis 8=other 9=unknown □ | | | | | | | | | |
| **D16.** Disease of pancreas | 0=No 1=Yes year | Treatment | 0=No 1=Yes（ year month） | 0=No 1=Yes | □ | □ | □ | □ | □ | □ |
| **D16a.** If yes | （Optional）：1= acute pancreatitis 2= chronic pancreatitis 8=other 9=unknown □ | | | | | | | | | |
| **D17.**  Gastrointestinal disease | 0=No 1=Yes year | Treatment | 0=No 1=Yes（ year month） | 0=No 1=Yes | □ | □ | □ | □ | □ | □ |
| **D17a.** If yes | （Optional）：1= gastric ulcer 2= duodenal ulcer 3= gastritis 4= chronic colitis 5= gastroesophageal reflux disease 6= constipation  8=other 9=unknown □ | | | | | | | | | |
| **D18.** Dermatosis | 0=No 1=Yes year | Treatment | 0=No 1=Yes（ year month） | 0=No 1=Yes | □ | □ | □ | □ | □ | □ |
| **D18a.** If yes | （Optional）：1= eczema 2= contact dermatitis 3=urticaria 4= psoriasis 5= vitiligo 8=other 9=unknown □ | | | | | | | | | |
| **D19.** Arthrolithiasis | 0=No 1=Yes year | Treatment | 0=No 1=Yes（ year month） | 0=No 1=Yes | □ | □ | □ | □ | □ | □ |
| **D20.** Tuberculosis | 0=No 1=Yes year | Treatment | 0=No 1=Yes（ year month） | 0=No 1=Yes | □ | □ | □ | □ | □ | □ |
| **D21.** How many times do you see doctor in the last year? ; How many times do you be hospitalized in the last year? . | | | | | | | | | | |
| **D22.** Have you had any illness in the last two weeks? 0=No（go to ***D23***） 1=Yes □ | | | | | | | | | | |
| **D22a.** 1= No treatment, no self-medication or adjunct therapy 2= No visit, but self-medication or some adjunct therapy 3= Go to a medical and health unit □ | | | | | | | | | | |
| **D23.** Have you taken any medications in the last two weeks? (optional) 0=No 1=Yes | | | | | | | | | | |
| 1= acesodyne 2= hypnagogue 3= antitussive 4= diuretic 5= asthma drug 6= thrombolytics 7= calcium supplement 8= antibiotic  9= hormone drug 10= anticoagulant 11=vitamin （1 compound vitamin 2 vitamin D 3 other ） 12= Aspirin（ year month） 13=other | | | | | | | | | | |

| **E、Physical activities** |
| --- |
| **E1.** Are there any difficulties in moving around autonomously? □  1=No 2=little 3=moderate 4= serious 5= can't walk |
| **E2.** Are there any difficulties in self-care (washing face, brushing teeth, dressing, going to the toilet, etc.)? □  1=No 2=little 3=moderate 4= serious 5= can't self-care |
| **E3.** Are there any difficulties in daily activities (work, reading or housework)? □  1=No 2=little 3=moderate 4= serious 5=can not |
| **E4.** Are you feeling any pain or discomfort today? □  1=No 2=little 3=moderate 4= serious 5= extreme |
| **E5.** Are you feel anxiety (such as nervousness, worry, unease, etc.) and / or depression (such as lack of interest in doing things, etc.) today: □  1=No 2=little 3=moderate 4= serious 5= extreme |
| **E6.** If you give yourself a total score for today's health, what points do you score?  （With a full score of 100, the higher the score, the healthier）  ├---┼—-┼—-┼—-┼—-┼—-┼—-┼—-┼—-┼—-┤  0 10 20 30 40 50 60 70 80 90 100 |
| Investigator signature： Investigation end Time： |
| Please evaluate the quality of this survey 1=high 2=medium 3=low □ |

| **(F)Physical examination** |
| --- |
| **First Second**  **F1.** Height   cm cm（Accurate to 0.1 cm）  **F2.** Waist   cm cm（Accurate to 0.1 cm）  **F3.** Hip   cm cm（Accurate to 0.1 cm）  Investigator signature： |
| **F4.** Weight  Kg（Accurate to 0.1Kg）  **F5.** Fat % %（Accurate to 0.1%）  **F6.** Basal metabolism Kcal  **F7.** Visceral fat index  Investigator signature： |
| Left hand **/**  Right hand  **F8.**  First time   / Kg（Accurate to 0.1Kg）  **F9.** Second time   / Kg（Accurate to 0.1Kg）  **F10.** Third time   / Kg（Accurate to 0.1Kg）  **F11.** Dominant hand 1=left 2= right □  Investigator signature： |
| **F12.** Sit-in for 5 minutes, measure blood pressure and pulse (rest not less than 30 seconds between measurements):  **Resting pulse**  First time / mmHg  /min  Second time / mmHg  /min  Third time / mmHg  /min  Investigator signature： |
